# Stability Amidst Change in the Measurement of Implementation Fidelity Over Time

**DOI:** 10.1007/s11121-025-01864-1

**Published:** 2026-01-06

**Authors:** Sydni A. J. Basha, Qiyue Cai, Melanie M. Domenech Rodriguez, Abigail H. Gewirtz, Margrét Sigmarsdóttir, David S. DeGarmo, Melissa Uribe, Marion S. Forgatch

**Affiliations:** 1 Department of Psychology & Reach Institute, Arizona State University, Tempe, AZ USA; 2 Department of Psychology, Utah State University, Logan, UT USA; 3 Paul Baerwald School of Social Work & Social Welfare, Hebrew University of Jerusalem, Jerusalem, Israel; 4 School of Education, University of Iceland, Reykjavík, Iceland; 5 College of Education, University of Oregon, Prevention Science Institute, Eugene, OR USA; 6 Implementation Sciences International Inc., Eugene, OR USA; 7 Oregon Social Learning Center, 10 Shelton Mcmurphey Blvd, Eugene, OR 97401 USA

**Keywords:** Implementation, Fidelity, GenerationPMTO

## Abstract

Children’s mental health disorders are rising, underscoring the need to implement behavioral parent training (BPT) programs. However, wide variability in BPT effectiveness often reflects inconsistencies in implementation fidelity. This study examines test–retest reliability of the GenerationPMTO model’s Fidelity of Implementation Rating System (FIMP) over a 17-year period. Seven coders provided ratings of 34 video segments from families participating in the Marriage and Parenting in Stepfamilies (MAPS) intervention, coded at two time points (2004, 2021) using first and third iterations of the FIMP manual. Variance decomposition analyses determined how much variability in scores was attributable to the interventionist, the observational coder, the session, and the year the data were coded. Test–retest intraclass correlation coefficients (ICCs) examined reliability across FIMP domains (knowledge, structure, teaching, process, and overall). Therapist differences accounted for the largest variance (38.1%), followed by coders (14.1%) and session (10.7%). Year did not significantly contribute, indicating that FIMP revisions have not undermined earlier fidelity metrics. Reliability analyses showed acceptable-to-excellent ICCs (range = 0.73–0.92), supporting the comparability of historical and current ratings. These findings indicate that GenerationPMTO’s FIMP refinements maintain core fidelity metrics. By demonstrating stable fidelity data over time, the study bolsters confidence in both historical results and current coding practices. These outcomes reinforce the utility of long-standing training materials and support the use of stable fidelity tools in ongoing implementation and training contexts. Such synergy between fidelity measurement and adaptation fosters sustained program effectiveness across service contexts, allowing providers to align newer fidelity protocols with established best practices.

**Supplementary Information:**

The online version contains supplementary material available at 10.1007/s11121-025-01864-1.

## Introduction

Over the past few decades, children’s mental health disorders have risen markedly (Deng et al., 2023; Lebrun-Harris et al., 2022), with recent estimates suggesting that nearly one in five children in the USA experiences a mental, behavioral, or developmental disorder (Centers for Disease Control and Prevention, 2023). Left unaddressed, these issues can have enduring implications for functioning and mental health in adulthood (Nelson et al., 2020). Mental health disorders in childhood are also linked to a range of negative functional outcomes in adulthood, including substance use and poorer overall health (Copeland et al., 2021). As such, children’s mental health constitutes a significant public health concern with wide-ranging social and economic repercussions, necessitating population-level approaches for effective intervention (Doyle et al., 2022).

Despite their needs, many children lack access to adequate services due to a variety of social, perceptual, structural, and systemic barriers (Radez et al., 2021). Fortunately, parents are an effective intervention target, as their behaviors and skills are critical mediators of children’s emotional and behavioral health (Sanders et al., 2022) and are modifiable with behavioral intervention. Acknowledging the importance of parenting skills and the family system, evidence-based behavioral parenting training (BPT) programs have become a foundational pillar in efforts to support families and improve child outcomes, with a vast array of programs emerging from research over the past several decades (Teti et al., 2017). This has resulted in an abundance of BPTs with a wide range of targeted populations and disorders.

Despite their broadening use, a growing body of literature has identified variability in the documented effectiveness of BPT programs (e.g., Al Sager et al., 2024; Kaminski & Claussen, 2017; Kjøbli et al., 2023). While this variability may be attributable to a range of factors, including differences in family characteristics, provider experiences, and contextual challenges, concerns have increasingly focused on how programs are implemented in real-world conditions. Specifically, implementation fidelity is a frequently hypothesized explanation for diminished or inconsistent effects observed when BPT programs are delivered in community settings (Chambers et al., 2013), although direct tests of this mechanism remain limited in some studies. This observed variation is increasingly understood as reflecting differences in how interventions are implemented. As Durlak and DuPre (2008) note, fidelity and adaptation frequently co-occur in community-based implementations, and both can influence outcomes. This reinforces the need for fidelity measurement tools that are sensitive to provider-driven adaptations while reliably assessing core intervention components. Indeed, effectively translating existing BPT programs from research to community and practice settings will require overcoming persistent barriers to implementation (Akiba et al., 2021; Doyle et al., 2022), and this will only be possible through additional research aimed at understanding and expanding strategies for sustainment on a larger scale (Olswang & Prelock, 2015). Accordingly, continued research is needed to better understand when and how fidelity contributes to observed intervention outcomes and to ensure that fidelity measurement tools remain valid and stable as interventions evolve.

Achieving sustainment at scale necessitates closing the research-to-practice gap for BPT programs. As in many disciplines, what research has taught the field about best practices for BPTs has far outpaced what providers actually do, in-part due to conflicting priorities among stakeholders (e.g., program developers, researchers, providers; Buchanan et al., 2024). Therefore, the question remains how to get BPT programs to providers in unique community and practice settings efficiently and without compromising program effectiveness (Mazzucchelli & Sanders, 2010; Olswang & Prelock, 2015).

### Implementation Fidelity

One crucial point of intersection among these competing priorities is implementation fidelity, typically defined as the extent to which providers adhere to the delivery of the program as it was designed and intended (Durlak & DuPre, 2008). In the process of transporting BPT programs to community and practice settings, implementation fidelity is a well-known culprit for decay in program effectiveness and is one of the most common strategic targets when trying to improve implementation (Akiba et al., 2021). In theory, maintaining high implementation fidelity involves strict adherence to prescribed protocols and procedures, ensuring that every component of an intervention is executed as intended.

However, research on dissemination and implementation highlights the importance of balancing fidelity with adaptation. Empirical studies (e.g., Cohen et al., 2008; Seay et al., 2015) emphasize the necessity of working with and training providers to implement BPT programs in a way that maintains core components while allowing for flexibility to meet the needs of diverse families and contexts (Durlak & DuPre, 2008; Mazzucchelli & Sanders, 2010). More specifically, BPT programs are typically designed and tested within rigorously controlled research environments, in which high fidelity to the program must be achieved and monitored if improvements in intended outcomes are to be attributed to participation in the program. Building this rigidity into experimental trials, combined with screening out “ineligible” participants, is designed to minimize noise and confounds, but comes at the expense of ecological validity and generalizability (Chambers et al., 2013) for both the providers and the intended population that developers and researchers hope will use the program after efficacy has been established.

As such, there have been substantial efforts to build flexible or adaptive fidelity into both program manuals and measures used to monitor fidelity (Kendall & Frank, 2018; Mazzucchelli & Sanders, 2010; Sanders, 2023). Among the measures that have demonstrated methodological reliability and predictive validity, fidelity often appears as a combination of skills related to provider’s intervention knowledge, adherence to the intended delivery (e.g., delivery as intended, dosage, and implementing necessary modifications), and competence or quality in delivery. Measuring fidelity in this comprehensive, component-level way includes skills related to the provider’s ability to adapt to the parents they serve and includes skills related to teaching, delivery process skills, rapport-building behaviors, session structure, effective use of physical materials, and maintaining goal/objective orientation (Basha et al., 2024). Despite efforts to capture how fidelity is defined and related to outcomes resulting from BPT programs, very little work has critically examined the robustness of the field’s fidelity measurement tools—that is, their ability to preserve measurement consistency without sacrificing sensitivity to clinically meaningful adaptation.

### Fidelity Monitoring in GenerationPMTO Parenting Programs

Among the array of available BPT programs, GenerationPMTO (formerly known as the Parent Management Training—Oregon Model; Rains et al., 2021) stands out not only in its demonstrated efficacy and effectiveness in improving positive parenting practices (Cai et al., 2022) but also for its commitment to rigorous fidelity monitoring (Sigmarsdóttir et al., 2023). Indeed, GenerationPMTO is an example of a widespread BPT program that has ensured an emphasis has been placed on effective, consistent fidelity monitoring throughout its global implementation efforts (e.g., Askeland et al., 2019; Forgatch & DeGarmo, 2011; Sigmarsdóttir et al., 2019). This structured approach has been instrumental in GenerationPMTO’s successful transition from research trials to implementation in real-world settings (Forgatch et al., 2013; Tommeraas & Ogden, 2017).

Despite these strengths, critical questions remain regarding the robustness and stability of GenerationPMTO’s fidelity measurement procedures over time. As individual programs continue to grow and expand, it is essential to understand how the measurement of fidelity has changed and what factors, if any, contribute to observed variability in both fidelity ratings and parent and child outcomes. Fidelity is a complex construct and the operationalizations and methods for monitoring and measuring provider fidelity across various BPT programs are inconsistent (Basha et al., 2024; Martin et al., 2023). A recent systematic review of fidelity illustrates how the field has evolved and subsequently developed a more precise understanding of the various factors that contribute to the construct of fidelity (Basha et al., 2024). This review also raisedquestions regarding prior findings of internal and predictive validity based on historical fidelity rating systems. Martin and colleagues (2021) systematically reviewed available instruments used to measure provider competent adherence in parenting programs, emphasizing that precise measurement is essential for capturing nuanced aspects of provider performance. The authors found that while a range of tools exist to measure fidelity, there are many that fall short in terms of available evidence for reliability, validity, and sensitivity, highlighting significant gaps in measurement rigor. This review underscores the need for the development of more rigorous and finely tuned instruments to improve the assessment of program delivery and ultimately strengthen intervention outcomes.

### Evolution of the Fidelity of Implementation Rating System (FIMP)

The defining feature of GenerationPMTO’s rigorous fidelity monitoring system is the Fidelity of Implementation Rating System (FIMP; initially described in Forgatch et al., 2005a, 2005b). A detailed description of the FIMP rating system is available in the Supplemental Materials. The first iteration established an observational system to evaluate how well GenerationPMTO providers delivered the intervention’s core components (Knutson et al., 2003). The FIMP rating system assessed provider performance across five core domains: *knowledge* of the model, session *structure*, *teaching* skills, *process* skills, and *overall* quality. Domains were assessed on a nine-point scale (i.e., 1–3 = *Needs Work*, 4–6 = *Acceptable*, 7–9 = *Good Work*) coded from 10 to 12-min segments of video recordings of sessions. The first iteration of the FIMP system demonstrated predictive validity, with higher fidelity scores associated with greater improvements in parenting practices and child outcomes (Forgatch et al., 2005a, 2005b; Hukkelberg & Ogden, 2013). This early version laid the groundwork for fidelity assessment by combining rigorous behavioral coding with an emphasis on capturing the nuanced process of change.

The FIMP manual was subsequently revised in 2009, built on the experiences gained from diverse implementation sites and international applications (Knutson et al., 2009). This update focused on enhancing reliability, clarifying the rating guidelines to better support the training of new raters, while attempting to make minimal changes so as not to disrupt the comparability of previously scored sessions. This second iteration preserved key objectives (e.g., improved consistency and ease of use) while integrating practical insights from the real-world applications of both the intervention’s implementation and need for fidelity monitoring. Importantly, this version of the FIMP rating system continued to demonstrate predictive validity (Forgatch & DeGarmo, 2011).

More extensive updates were included in the FIMP rating system’s third iteration, following the rebranding of the intervention to GenerationPMTO (Knutson et al., 2019). This revision consolidated components: for example, assessment of role-plays conducted with parents to teach and practice skills was moved entirely into the domain assessing teaching skill. Additionally, the domain previously called “overall quality” was renamed “overall development” to better capture its intended focus on assessing a provider’s ability to engage families in the change process. This change corrected earlier misconceptions that the domain simply averaged other scores, instead emphasizing its role in recognizing contextual sensitivity, such as positive family changes despite challenges like parent–child separation, ongoing divorce, or legal mandates for participation. In this third iteration, language throughout the manual was streamlined for clarity and consistency, with added emphasis on cultural and contextual considerations.

Specifically, enhanced rating guidelines, clearer examples, and revised cues were incorporated to improve clarity in which domain-specific provider behaviors should be rated. Among these revisions, a specific emphasis was placed on distinguishing iatrogenic confrontational interactions that should be rated as (ineffective) teaching behaviors versus those that should be rated as (effective) process-related behaviors impacting provider-parent rapport. For example, a provider stating “Yes, but you aren’t doing it correctly. Don’t you remember last week when I told you to do…” would be rated low in the teaching domain due to the emphasis on trying to over-correct the parent’s knowledge, while a provider stating “Why would you do that? Do you want your child to continue to misbehave?” would be rated low in the process domain. These iterative refinements have collectively advanced the FIMP rating system to assess provider fidelity across diverse research and practice settings. Despite this theoretical increase in conceptual precision, no study to date has assessed putative variability in fidelity ratings from iteration to iteration.

### The Current Study

This study will enhance understanding of the precision of fidelity measurement—or its ability to consistently detect meaningful, provider-specific differences in implementation quality while minimizing error introduced by raters or measurement conditions—through two primary aims. First, *Aim 1* elucidates the etiology of existing variation in fidelity ratings. To do so, this study uses variance decomposition analyses to determine the proportion of the variance in fidelity ratings over time that may be attributed to the therapist delivering the intervention, the observational rater coding the fidelity, the intervention session, and the year the rating was coded (representing which iteration of the FIMP rating system was used).

*Aim 2* will examine the robustness of GenerationPMTO’s fidelity rating procedures by evaluating the reliability of scores coded with the first iteration of the FIMP rating system (Knutson et al., 2003) versus scores coded with the most recent iteration (Knutson et al., 2019). To determine this, analyses assessed reliability (utilizing intraclass correlation coefficients [ICC]) in fidelity ratings across time. Additionally, given prior research demonstrating the predictive validity of the FIMP rating system (e.g., Forgatch & DeGarmo, 2011; Forgatch et al., 2005a; Hukkelberg & Ogden, 2013), this study examined correlations between fidelity ratings from past and current iterations, with particular attention to potential differences across the five domains of the FIMP rating domains: knowledge, structure, teaching, process, and overall.

Through these aims, this study will provide evidence of the stability and persistence of fidelity criteria over time, despite changed and theoretically upgraded, more specific measurement tools. Insights from these analyses will have significant implications for the continued refinement of fidelity measurement, as researchers and providers alike undertake efforts to reduce the burden associated with rigorous fidelity monitoring as implementation is expanded. A deeper understanding of fidelity dynamics may ultimately enhance the scalability and sustained effectiveness of BPT programs in a variety of real-world settings.

## Method

### Procedures

#### Marriage and Parenting in Stepfamilies (MAPS) Program

The current study draws on data from the MAPS (Marriage and Parenting in Stepfamilies) program, a GenerationPMTO-based program adapted specifically to address the needs of stepfamilies. MAPS is a 13-session program designed for stepfamilies with a biological mother and stepfather. Extensive information about the intervention has been documented elsewhere (e.g., Bullard et al., 2010; Forgatch et al., 2005b; Wachlarowicz et al., 2012). Briefly, session agendas included objectives and rationales, practice exercises, role-plays, and troubleshooting. To support learning at home, parents receive materials that summarize the key principles of the intervention, along with home practice assignments, incentive charts, and other required materials. Although the manual provided a fixed session framework, interventionists were encouraged to modify the session structure or presentation of the materials to accommodate the context brought by each unique family.

The sessions were centered on the five core parenting skills of GenerationPMTO including skill encouragement, discipline, monitoring, problem solving, and positive involvement. Specialized content was included for the unique experiences of stepfamilies, such as presenting a united parenting front and clarifying the stepparent role. Overall, sessions are delivered in a progressive and integrated manner, with content iteratively building. Briefly, content included issues specific to stepfamilies, communication, problem-solving, effective directions, positive teaching techniques, reinforcement, encouragement, noncoercive discipline and limit setting, monitoring children in various settings, and reinforcing positive school-related behaviors.

#### Coders and FIMP Training

A total of seven coders provided FIMP ratings in either 2004 (*n* = 1), 2021 (*n* = 4), or in both years (*n* = 2). All coders were extensively trained in delivering GenerationPMTO, coaching and supervising others delivering the intervention, and coding fidelity with the FIMP rating system. Indeed, all seven coders are either developers of the coding system or extensively trained fidelity coders, certified by Implementation Sciences International, Inc. (ISII; the non-profit that manages global training and implementation of GenerationPMTO). The FIMP certification process requires prior certification in GenerationPMTO delivery, followed by over 40 h of training in the FIMP rating manual. Trainees learn to apply the manual to video-taped segments and must pass a test by achieving an intraclass correlation coefficient (ICC) greater than 0.70 to demonstrate adequate reliability. Then, maintaining reliability and preventing inter-rater drift are managed within each GenerationPMTO site’s individual fidelity team, and ISII-certification is maintained through a yearly reliability test that requires submission of a minimum of four “test spots” determined by the ISII fidelity consensus team.

### Participants

The original study included *N* = 110 families with recently married biological mothers and stepfathers, all recruited from a large metropolitan area of the Pacific Northwest. Specific information about the sample has been extensively reported elsewhere (e.g., Forgatch et al., 2005b). Briefly, couples had been married for an average of 15.58 months (*SD* = 12.56). Target children were the mothers’ biological child, living with her at least half of the time. Mothers and fathers reported average ages of 31.3 and 32.7 years (*SD* = 5.37 and 6.60), respectively. Target children were, on average, 7.47 years (*SD* = 1.15). The intervention was delivered by a team of four providers who held advanced degrees (two with master’s degrees and two with doctorates). Among them, two had prior experience as interventionists, while the other two were new to delivering GenerationPMTO. All four providers received training from the intervention developer and received subsequent supervision and training throughout the study’s implementation.

### Measures

#### Video Segments

FIMP segments selected for coding were drawn from sessions focused on three sessions in which critical intervention components—effective directions, skill encouragement, and discipline—are introduced to parents. Segments, also called “spots,” refer to a meaningful portion of a session, ideally lasting approximately 10 min (range of 9 to 12 min) during which the core topic of the session is thoroughly covered. Segments were selected by one of the original rating system developers to ensure they contained a continuous period that offers ample material for evaluating all five FIMP domains. Effective segments are typically found during role-plays, practice exercises, or while the provider explains or demonstrates the session topic, although the presence of a role-play is not mandatory for selection. Segments are either drawn from introductory or troubleshooting sessions. In introductory sessions, the provider explains the core parenting skill, demonstrates a specific strategy for using the skill, uses role-play to practice the method, and then reviews and summarizes the concept. In troubleshooting sessions, the focus is addressing challenges with home practice, tailoring the skill to fit family needs, reviewing and practicing methods, and integrating the skill with other components of the intervention. For example, troubleshooting encouragement might involve refining an incentive system or role-playing giving a child praise, while troubleshooting discipline might include role-plays to improve techniques to give a time out or brainstorming alternative strategies.

#### FIMP Coding

In 2004, fidelity ratings were obtained utilizing the first edition of the FIMP coding manual (Knutson et al., 2003). Coded intervention sessions included *n* = 34 video segments drawn from *n* = 16 families randomized to the intervention arm of the MAPS trial. All segments were coded by MSF, the intervention developer, and double coded by one of two additional coders. All three coders were the original developers of the FIMP coding system. In 2021, fidelity ratings were obtained utilizing the third edition of the FIMP coding manual (Knutson et al., 2019). The same *n* = 34 video segments from *n* = 16 families coded in 2004 were re-coded. All sessions were coded by MSF and two or more of six additional certified coders and members of the ISII fidelity team.

### Data Analyses

Variance decomposition models were guided by generalizability theory (G Theory; Mushquash & O’Connor, 2006) and were used to decompose the variance into person (p), item (i), and coder (c) components, specifically including variance attributable to the therapist delivering the intervention, the observational rater coding the fidelity rating, the session of the intervention, and the year that the spot was coded (i.e., 2004 vs. 2021). Then, test–retest interrater intra-class correlation analyses (ICC) were conducted between 2004 FIMP coding and 2021 FIMP coding for all five fidelity domains, as well as the sum score. ICC values were categorized following guidance from Koo and Li (2016), with categories including poor (< 0.50), moderate (0.50–0.75), good (0.75–0.90), and excellent (> 0.90). All analyses were done in IBM SPSS Version 29.0.2.0 (IBM Corporation, 2023).

## Results

### Variance Decomposition Analyses

In total, 745 individual observed ratings were included in the variance decomposition analysis, covering the five fidelity dimensions outlined in the manuals: knowledge, structure, teaching, process, and overall. Ratings were coded at two time points (2004 *n* = 340, 2021 *n* = 405) and were provided by the seven FIMP coders (range = 35, 168 videos per coder; with MF [the reliability coder] coding 325 videos). The ratings assessed the performance of the four providers that delivered the MAPS intervention across three content sessions (*n* = 25 segments for effective directions; *n* = 385 segments for discipline; *n* = 335 segments for encouragement).

**Fig. 1:**
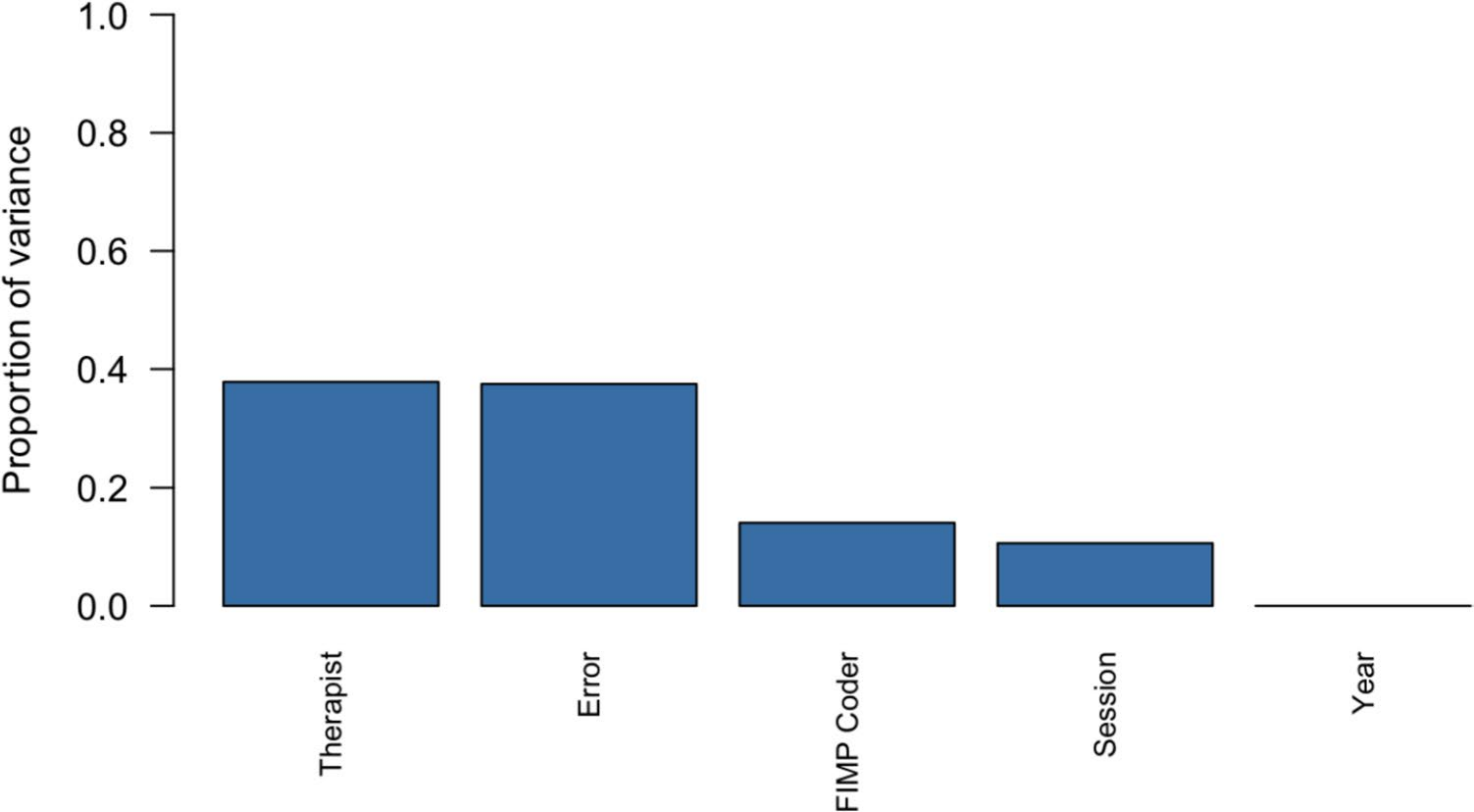
Bar plot of variance contributed by therapist, coder, session, year, and other/error

Main effects in the variance decomposition analyses with restricted maximum likelihood estimation were used to evaluate independent contributions of each facet to the total variances observed in the 745 rated sessions. Those facets included the therapist, the FIMP coder, the session, and the year. One-way ANOVA was used to evaluate whether each facet contributed significantly to the variance in fidelity ratings (Fig. 1). In this context, therapist-level variance reflects the degree to which fidelity scores differed based on who delivered the intervention; coder-level variance indicates differences in how individual FIMP raters assigned scores; session-level variance captures the influence of the specific session being rated; and year-level variance reflects the use of different FIMP manual versions (2004 vs. 2021). As hypothesized, the results revealed that the largest proportion of variance was attributed to differences between therapists delivering the session (38.1%, *F* (3, 732) = 231.92, *p* < 0.001). Other significant contributors to the observed variability included different FIMP coders (14.1%, *F* (6, 732) = 32.54, *p* < 0.001) and the intervention session (10.7%, *F* (2, 732) = 64.04, *p* < 0.001). Coding year did not significantly contribute to the observed variance (0%, *F* (1, 732) = 1.51, *p* = 0.220) in fidelity ratings.

When including interactions between the therapist, the FIMP coder, and the year, the results showed a significant three-way interaction (*F* (1, 732) = 4.62, *p* = 0.003); however, it only explained an additional 2.20% of the total variance, and the significance was likely due to different FIMP coders contributing to the ratings in 2004 versus 2021. The interaction between the therapist and the FIMP coder significantly contributed to the total observed variance, (39.7%, *F* (13, 714) = 14.32, *p* < 0.001). The interaction between the therapist and the year the video was coded was significant (*F* (3, 714) = 4.97, *p* = 0.002), and post hoc analysis revealed that one therapist was rated significantly higher in 2004 than in 2021.

### Reliability and Correlation Analyses

In total, 68 session ratings and 28 aggregated unique family-session-therapist ratings were available in 2004 while 81 session ratings and 27 aggregated unique family-session-therapist ratings were available in 2021. To examine the stability of the fidelity coding system over time, these aggregated ratings were utilized to assess the reliability and correlations between ratings coded in 2004 and 2021, taking care to examine differences that may have emerged at the domain-level (Table 1).

**Table 1:** Means, standardized deviations, paired *t*-tests, and intraclass correlations (ICCs) between fidelity ratings

FIMP domain	2004 Rating		2021 Rating		Paired *t*-test		*r*	ICC (CI)
	*n*	*M* (*SD*)	*n*	*M* (*SD*)	*t*-value	*P*-value		
Knowledge	27	7.41 (0.89)	27	7.15 (0.93)	1.98	0.059	0.72	0.82 (0.61, 0.92)
Structure	27	7.26 (1.01)	27	6.86 (0.96)	2.41	0.023	0.61	0.73 (0.39, 0.88)
Teaching	27	6.86 (1.23)	27	6.61 (1.08)	1.68	0.105	0.78	0.86 (0.70, 0.94)
Process	27	6.68 (1.36)	27	6.77 (1.31)	−0.70	0.492	0.85	0.92 (0.82, 0.96)
Overall	27	6.94 (1.24)	27	6.76 (1.09)	1.17	0.254	0.77	0.86 (0.70, 0.94)
Average score	27	7.03 (1.09)	27	6.83 (1.01)	1.63	0.115	0.82	0.89 (0.77, 0.95)

*M* mean, *SD* standard deviation, *r* Pearson’s, *r* correlations, *ICC* intraclass correlation coefficient, *CI* confidence interval

Across all FIMP dimensions, comparisons between the 2004 and 2021 ratings generally showed medium-to-large correlations and strong interrater reliability, though some differences emerged. For *knowledge*, the correlation was medium-to-large (*r* = 0.72), there were no significant differences on the paired *t*-test (*t*(26) = 1.98, *p* = 0.059), and the two-way mixed effects model indicated good test–retest reliability (ICC = 0.82, 95% CI [0.61, 0.92]). In the *structure* domain, the correlation was also medium-to-large (*r* = 0.72), but the paired *t*-test revealed a significant difference (*t*(26) = 2.41, *p* = 0.023), with moderate reliability (ICC = 0.73, 95% CI [0.39, 0.88]). For *teaching*, there was a large correlation (*r* = 0.78), no significant differences on the paired *t*-test (*t*(26) = 1.68, *p* = 0.105), and good reliability (ICC = 0.86, 95% CI [0.70, 0.95]). The *process* dimension showed an even stronger correlation (*r* = 0.85), no significant differences (*t*(26) = −0.70, *p* = 0.492), and excellent reliability (ICC = 0.92, 95% CI [0.82, 0.96]). In the *overall* domain, ratings were highly correlated (*r* = 0.77), with no significant differences (*t*(26) = 1.17, *p* = 0.254), and good reliability (ICC = 0.86, 95% CI [0.70, 0.94]). Finally, the average FIMP scale score also displayed a large correlation between 2004 and 2021 (*r* = 0.82), showed no significant differences (*t*(26) = 1.63, *p* = 0.115), and achieved good reliability (ICC = 0.82, 95% CI [0.77, 0.85]).

## Discussion

This study provides important insights into how fidelity has been assessed within GenerationPMTO across time. Specifically, it demonstrates that FIMP coders using different versions of the manual arrived at similar conclusions about therapist performance, supporting the long-term consistency of the system. These findings are directly relevant to programs and researchers using FIMP and may also inform broader conversations about the evolution of fidelity systems over time. Against the backdrop of growing mental health needs among children and adolescents (Deng et al., 2023; Lebrun-Harris et al., 2022; Nelson et al., 2020), BPT programs have emerged as critical avenues for intervention (Sanders et al., 2022; Teti et al., 2017). However, consistent program effectiveness and the potential for population-level change hinge on high-quality implementation (Akiba et al., 2021; Doyle et al., 2022). The current findings suggest that, despite meaningful changes in GenerationPMTO’s FIMP rating system over time, older and newer fidelity scores align sufficiently to allow for valid comparisons across iterations. This is a crucial finding for a field in which maintaining consistent measurement standards is essential for interpreting fidelity over time, particularly as programs adapt to real-world settings (Chambers et al., 2013).

One of the key challenges in disseminating BPT programs at scale is balancing adherence to the intervention’s core components with the flexibility needed to serve diverse families. Variance decomposition analyses revealed that the year in which fidelity was coded (i.e., which iteration of the FIMP manual was used) did not significantly contribute to observed variability in scores, suggesting that changes in the FIMP rating system over time have not destabilized its core measurement properties. Instead, the greatest sources of variation were the therapists themselves, followed by differences among coders and then the specific session being observed. This outcome reinforces the importance of developing fidelity measurement systems that are dynamic enough to capture real-time adaptations while preserving consistent rating criteria, which is a crucial prerequisite for sustaining confidence in the reliability of implementation across the course of delivery (Kendall & Frank, 2018; Mazzucchelli & Sanders, 2010). Notably, such flexibility supports the idea that BPT programs can evolve in-step with the contextual realities of community practice, essential for interventions implemented under diverse cultural, socioeconomic, and systemic conditions (Chambers et al., 2013; Olswang & Prelock, 2015).

Findings reinforce the need for fidelity measurement systems to evolve with real-world adaptations, an issue that is gaining renewed prominence in implementation science (Begeny et al., 2023; Ginsburg et al., 2021; Martin et al., 2024). As BPT programs expand into more varied and complex delivery formats and settings, further research is needed to refine fidelity tools in a way that systematically documents both planned and unplanned modifications, thereby providing clearer evidence on what truly drives program success. By ensuring robust fidelity measurement and dynamic adaptation are at the fore, all stakeholders can better ensure that evidence-based interventions remain both effective and responsive to the ever-changing realities of community-based practice.

The result that all FIMP domains displayed good test–retest reliability (ranging from acceptable to excellent) between the first and most recent coding manuals is particularly encouraging. It indicates that the fundamental criteria for assessing fidelity in GenerationPMTO have been preserved across iterations, thereby validating historical results that used the earlier version of the FIMP rating system (Forgatch et al., 2005a; Knutson et al., 2003). This continuity is vital for the numerous research groups and implementation practice sites that rely on existing training videos and archives of coded sessions to train new providers and re-certify experienced ones. Similarly, the certification of new FIMP coders heavily relies on the extensive archives of previously coded videos and consensus ratings. This study reinforces confidence that the use of these training materials still produces valid and reliable coders. Because re-coding entire libraries of videos each time a manual is updated is impractical and unfeasible, maintaining the practical utility of established historical materials is crucial. Furthermore, if historical ratings were invalid under new criteria, the field would either face a serious gap in its evidence base for BPT programefficacy or need to consider if existing measures are indeed accurately conceptualizing and capturing provider fidelity. Instead, these results support the stability of earlier findings, which have demonstrated predictive validity for child and family outcomes (Forgatch & DeGarmo, 2011; Forgatch et al., 2005a).

The modest but significant difference in the structure domain highlights that certain aspects of fidelity measurement may be more sensitive to updated coding guidelines. While overall reliability of the structure domain remained moderate, this fluctuation may reflect how changes in the FIMP manual refined operational definitions of what constitutes strong session structure. Specifically, this might have resulted in a cohort effect, wherein structure codes from 2021 were statistically significantly lower because expectations for what constitutes strong session structure (i.e., following an agenda, pacing the session well, transitioning smoothly between topics) became stricter with the third iteration of the manual.

Findings also shed light on the broader evolution of GenerationPMTO, wherein streamlined practices and clarifications of operational definitions have advanced in line with global implementation efforts (Askeland et al., 2019; Forgatch et al., 2013; Sigmarsdóttir, et al., 2019). By refining its fidelity rating system in tandem with program improvements, GenerationPMTO illustrates how fidelity measures can evolve to capture necessary real-world adaptations while still protecting measurement integrity. Indeed, as Martin and colleagues (2024) highlight, fidelity tools that ignore providers’ contextual realities risk overlooking nuances and inaccurately capturing provider performance. The updates made to the current edition of the manual represent precisely the sort of fine-grained distinctions that allow fidelity coders to parse out the nature of provider behavior more accurately (i.e., behaviors and decisions caused by lack of knowledge of the intervention versus those decisions made consciously and strategically to best support the family they are serving).

Moreover, these refinements reflect a growing recognition that interventions must adapt to families’ unique cultures, developmental stages, and needs, which can vary substantially. In line with calls to accommodate both fidelity and adaptability (Cohen et al., 2008; Mazzucchelli & Sanders, 2010; Seay et al., 2015), GenerationPMTO’s approach demonstrates that it is possible to formalize a rigorous measurement system that encourages providers to tailor sessions while remaining grounded in evidence-based practices. The current study thus provides an empirical basis for trusting the comparability of FIMP ratings across multiple iterations, which is critical as GenerationPMTO continues to expand into new contexts. These findings hold the most immediate relevance for implementation scientists and practitioners who use the FIMP system in GenPMTO-based programs. In particular, they may offer reassurance to trainers, fidelity monitors, and program developers that historical fidelity data remain interpretable and useful in understanding implementation processes over time. However, more broadly, this work may be informative to researchers and practitioners developing fidelity tools for complex, multi-component interventions that require long-term sustainability.

### Strengths, Limitations, Implications, and Future Directions

Despite being the first study, to our knowledge, to demonstrate stability across multiple decades of fidelity assessment efforts, this work was not without its limitations. Although adapting an implementation strategy can bolster ecological fit, it also raises the question of how best to track fidelity without imposing excessive burden on providers and researchers. Recent research highlights multiple avenues for addressing this dilemma. For instance, mixed methods fidelity assessments that combine self-report, observation, and qualitative feedback can capture essential contextual information about why, how, and when adaptations occur (Akiba et al., 2021). Additionally, preliminary work assessing the accuracy and feasibility of utilizing automated or technologically assisted methods are promising. For example, machine learning–based natural language processing models have demonstrated initial validity in coding fidelity in other family-based preventive interventions (Berkel et al., 2023; Gallo et al., 2021). These novel approaches align with the findings of the current study; even when the overarching measurement tool evolves, the key is to preserve core fidelity constructs, building in enough flexibility to accurately represent required changes that occur in the therapy room with families, and attempt to reduce burden on providers and researchers.

Additionally, these findings are naturally somewhat restricted to the GenerationPMTO family of BPT programs, given that the FIMP rating system is only used for programs based on this model. However, other evidence-based BPT programs are scaling up for use at the population level and working to practically integrate efficient and effective fidelity monitoring tools. As programs move from research to practice settings, adjustments to their fidelity monitoring systems may become necessary and the results of this study should lend confidence that alterations are possible without disturbing core assumptions. Despite this narrow scope, results illustrate that older training resources can still be effectively utilized alongside updated FIMP manuals, offering practical value for organizations by maintaining compatibility between legacy materials and updated fidelity manuals. However, future research should replicate these findings in additional BPT contexts to determine whether similar stability in fidelity assessments holds when different theoretical models, populations, or adaptations are involved.

Nevertheless, our results highlight the ongoing importance of calibrating coders to ensure consistency, given that coder differences contributed notably to the variance. Future studies could delve deeper into the coder-level characteristics that shape fidelity judgments, such as clinical background, length of experience, or training protocols. Similarly, research might explore whether certain fidelity dimensions are more prone to drift over time. Future work should more systematically evaluate how specific coder-level attributes influence ratings, potentially informing targeted training modules or credentialing processes to further standardize fidelity assessments. Additionally, continued refinement of fidelity measures should incorporate advanced data collection and analytics, potentially leveraging technology (e.g., automated coding tools or machine learning, e.g., Berkel et al., 2023; Gallo et al., 2021) to expedite fidelity checks and reduce the burden on implementation partners (Basha et al., 2024). Such methods could help reduce coder bias, enhance reliability, and further streamline the fidelity monitoring process for new implementation sites.

Finally, this study was not sufficiently powered to examine how the predictive validity of the newest iteration of the FIMP manual compares to prior versions, which limits the ability to draw conclusions about the ultimate benefit of consistent fidelity measurement. Even so, by establishing the robustness of historical data, this study has laid the foundation for future investigations that may link updated fidelity scores to long-term results. Future research should take care to examine the relationship between fidelity and child or family outcomes, particularly in marginalized communities where changes may be necessary to address unique cultural or systemic barriers.

### Conclusion

In sum, these results demonstrate that the FIMP rating system, despite undergoing multiple iterations, remains a reliable measure that has demonstrated consistency over time in assessing fidelity in GenerationPMTO. Despite 17 years between fidelity ratings, the minimal impact of year (i.e., the iteration of the FIMP manual) on the variance in fidelity scores offers strong evidence for the robustness of the GenerationPMTO fidelity measurement (Forgatch & DeGarmo, 2011; Forgatch et al., 2005a; Hukkelberg & Ogden, 2013). This study thereby affirms that fidelity measurement can evolve to accommodate both rigorous adherence and the practical adaptations required for real-world application. As the prevalence of childhood mental health disorders continues to rise (Copeland et al., 2021; Deng et al., 2023; Lebrun-Harris et al., 2022), ensuring that BPT programs like GenerationPMTO remain both flexible and evidence-based is essential for scale up to meet urgent public health needs. By confirming the stability of prior fidelity data, this study supports the continued use of earlier fidelity scores in provider training and certification, helping to reduce resource demands and enhance implementation feasibility. Ultimately, this strengthens the potential for sustained, high-quality implementation of interventions aimed at promoting better outcomes for children and families in diverse contexts.

## Supplementary Information

Below is the link to the electronic supplementary material.

ESM 1 (PDF 105 KB)

## Data Availability

The data reported on here are available from the corresponding author upon request.
