# Supplementary material for: Stability Amidst Change in the Measurement of Implementation Fidelity Over Time
Source: Prev Sci. 2026 Jan 6;26(8):1251–62. doi: 10.1007/s11121-025-01864-1 (PMC12804281; doi:10.1007/s11121-025-01864-1)
Supplement: Supplementary file 1 — (PDF 105 KB) [file 11121_2025_1864_MOESM1_ESM.pdf]

Supplemental Material: Overview of the Fidelity of Implementation Rating System (FIMP)

The **Fidelity of Implementation Rating System (FIMP)** is a structured observational coding system developed to assess provider fidelity to the GenerationPMTO model (formerly known as the *Parent Management Training-Oregon Model [PMTO])*. Fidelity, in this context, refers not only to adherence to the program's content and structure but also to the skill, responsiveness, and engagement strategies providers use during intervention delivery. FIMP is an empirically established tool for rating fidelity in the GenPMTO family of interventions and has undergone extensive validation, including associations with provider training success and downstream family outcomes.

FIMP ratings are based on the observation of videotaped or live-delivered PMTO sessions and are assigned by trained raters. The system assesses five core dimensions of provider behavior: **Knowledge, Structure, Teaching, Process Skills, and Overall Development**. Each is rated on a 9-point scale where 1–3 = *Needs Work*, 4–6 = *Acceptable*, and 7–9 = *Good Work*. Ratings incorporate specific provider actions and interactions across multiple behavior categories within each dimension.

Note that the following lists of behavioral indicators are not exhaustive and are meant to give our interested readers an idea of what components go into each domain.

1. GenPMTO Knowledge

This dimension assesses how accurately and fluently the provider applies GenPMTO principles and strategies throughout the session. It reflects both their technical mastery and their ability to integrate the core concepts of the intervention model into session content.

Key elements considered in this rating include whether the provider:

- Uses PMTO-specific language and procedures correctly
- Demonstrates sound understanding of the parenting tools being taught
- Applies the principles and model while avoiding drift into unrelated or unstructured content
- Seamlessly integrates the intervention principles into the session in a developmentally appropriate and context-sensitive manner

A high score reflects precise use of GenPMTO strategies in real time, demonstrating both theoretical knowledge and flexible application.

2. Structure

The Structure dimension reflects how effectively the provider organizes and manages the session. This includes how well they prepare, pace, and lead the conversation to ensure the session unfolds logically and efficiently.

Key observable behaviors include whether the provider:

- Follows the session agenda and maintains appropriate flow
- Includes all relevant appropriate sections of the material and does not leave out critical components
- Transitions smoothly between appropriate sections while summarizing covered materials
- Manages time effectively and avoids getting stuck or distracted
- Maintains leadership of the session while remaining attuned and responsive to family needs

Sensitive pacing, non-dominating leadership, and the ability to maintain session direction without becoming overly rigid or overly passive are hallmarks of strong performance in this domain.

3. Teaching

Teaching captures the provider's skill in actively promoting learning and comprehension of parenting strategies. It focuses on how well the provider delivers content and facilitates engagement with key parenting tools.

This domain includes a wide range of behaviors, such as:

- Presenting information clearly and concisely
- Using a variety of teaching techniques, such as modeling, visual aids, and chunking content into teachable units
- Eliciting participation from caregivers through questions and guided dialogue
- Monitoring for understanding and adapting when necessary
- Providing clear rationales for each parenting practice introduced
- Offering encouragement and carefully constructive correction as families practice new skills

Teaching also includes the use of role plays to enhance experiential learning. Indeed, role plays are considered critical to effectively teaching skills to parents. High-quality teaching fosters both skill acquisition and confidence in use.

4. Process Skills

Process Skills refers to how effectively the provider manages the therapeutic interaction and communication process. This includes not only the techniques used to deliver content but also the interpersonal and relational dimensions of the session.

Key aspects include whether the provider:

- Prevents or manages conflict in session
- Uses appropriate questioning strategies to promote reflection and participation (i.e., asks questions that elicit parents' learning and/or review of prior learning)
- Responds to emotional content and tailors the approach to each family's needs
- Encourages problem solving, accountability, and active involvement from parents

- Shows empathy and maintains a balance between structure and support
- Uses foundational therapeutic skills where appropriate (e.g., reinforcement, normalization, validation, paraphrasing, reflections, reframing, mirroring, etc.)
- Connects to the family's storyline and individual circumstances

Additionally, the use of PMTO-specific tools (e.g., "the tool menu") is assessed here in terms of appropriateness, accuracy, and timing. Strong process skills ensure that families feel heard, respected, and empowered.

5. Overall Development

Rather than duplicating the other domains, the Overall Development domain reflects the integration of multiple competencies, as well as the family's engagement and observed impact.

The Overall Development rating considers:

- The extent to which the provider demonstrated GenPMTO fluency and effectiveness across domains
- The likely impact of the session, including the family's responsiveness and participation in session, and consideration for whether it appears the family is likely to attempt to implement the skills at home
- Evidence of parent satisfaction, growth, and buy-in (e.g., verbal enthusiasm, future orientation, or increased confidence)
- Provider's ability to individualize delivery (i.e., their ability to consider and adapt to individual family's unique contexts) without compromising adherence to program guidelines

Importantly, this domain is where raters incorporate judgment about whether the provider balanced adherence with adaptability and connection.

Rating System and Use

Each of the five domains is rated independently, and scores across dimensions are used to generate profiles of provider fidelity over time. Raters undergo extensive training and calibration to ensure reliability and reduce bias. Ratings are often used to support provider certification, provide feedback during training, and monitor implementation quality during intervention trials or scale-up efforts.
